# Plasma-derived exosomal pyruvate kinase isoenzyme type M2 accelerates the proliferation and motility of oesophageal squamous cell carcinoma cells

**DOI:** 10.3892/or.2022.8256

**Published:** 2022-01-04

**Authors:** Lifei Yang, Shutao Zheng, Qing Liu, Tao Liu, Qiqi Zhang, Xiujuan Han, Aerziguli Tuerxun, Xiaomei Lu

Oncol Rep 46: Article no. 216, 2021; DOI: 10.3892/or.2021.8167

Subsequently to the publication of the above article, the authors have realized that Fig. 5B on p. 8 was compiled erroneously, in the sense that the two immunohistochemical images selected for Fig 5B did not correspond to each other, meaning they were not derived from the same field under the microscope. This error was inadvertently made during the preparation of the manuscript.

A corrected version of Fig. 5, showing the correct data for the expression of PKM2 in NAT in Fig. 5B, is shown on the next page. This inadvertent error did not affect the conclusions reported in this paper, and all the authors agree with this Corrigendum. The authors sincerely thank the Editor of *Oncology Reports* for presenting them with the opportunity to publish this Corrigendum, and apologize to the readership of the journal for any inconvenience caused.

## Figures and Tables

**Figure 5.**
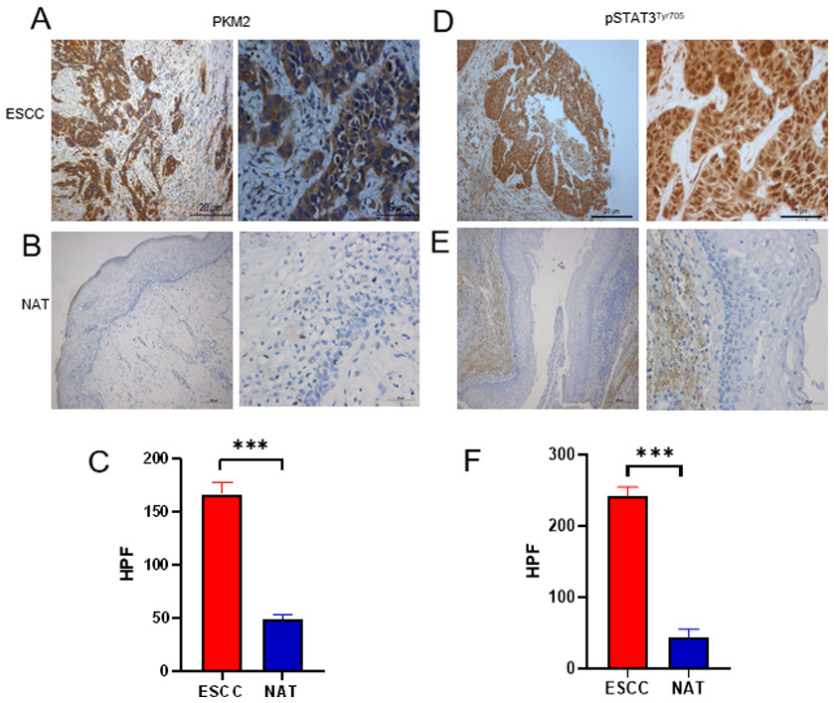
PKM2 and pSTAT3Tyr705 is highly expressed in ESCC tissues. Representative images of immunohistochemical staining of PKM2 in (A) ESCC and (B) matched NAT. (C) Quantitative analyses of immunohistochemical PKM2 data. Representative images of the immunohistochemical staining of pSTAT3Tyr705 in (D) ESCC and (E) NAT. (F) Quantitative analyses of immunohistochemical data on pSTAT3Tyr705. ***P<0.001. Magnification, ×100 and ×400; scale bar, 20 and 5 µm, respectively. ESCC, oesophageal squamous cell carcinoma; PKM2, pyruvate kinase isoenzyme type M2; HPF, high power field; NAT, normal-adjacent tissue.

